# Has the green total factor productivity increased in the early stage of the establishment of smart city

**DOI:** 10.1371/journal.pone.0322922

**Published:** 2025-05-22

**Authors:** An-wei Wan, Wei Cui

**Affiliations:** 1 Business School, Hohai University, Nanjing, R.P. China; 2 Business School, Nanjing Institute of Technology, Nanjing, R.P. China; Universidade de Lisboa Instituto Superior Tecnico, PORTUGAL

## Abstract

In the context of global climate change, green development has become the main goal of smart city construction. Most existing research suggests that smart cities will enhance the level of the green total factor productivity (GTFP) in cities. However, this study found that smart cities will reduce the level of green total factor production in the short term and increase it in the long term. Based on this, this article selects three batches of smart cities in China from 2013 to 2019, and uses the Malmquist index model, common frontier function, and panel data method to analyze the GTFP model in the early stage of smart city construction in China. The study found that: (1) the GTFP of the three batches of smart cities in the early stage of construction was less than 1 and showed a downward trend, indicating that smart cities will reduce the GTFP level of cities in the short term. (2) Technical efficiency is the main reason for the decline of GTFP in the early stage of smart city construction and the rise of GTFP in the medium and long term. Specifically, there is a U-shaped relationship between the technological efficiency of smart cities and their GTFP. For every 1% increase in technical efficiency in the later stages of smart cities, GTFP increases by 47.3%. (3) The GTFP in the process of smart city construction shows a trend of decreasing in the early stage and increasing in the middle and later stages. The GTFP level in the later stage of smart cities is greater than 1 and shows a fluctuating upward trend, indicating that smart cities will improve the city’s GTFP level in the long run. In view of this, we should attach importance to ecological protection in the early stage of smart city construction and take effective measures to reduce carbon emissions during this period. During this period, policies such as taxation can be implemented to encourage companies to adopt cleaner production technologies, strengthen the exchange of green technologies between cities, accelerate the flow of green knowledge, reduce redundant construction of information infrastructure, and thus minimize the decline in GTFP in the early stages of smart city construction. This study provides policy recommendations and decision-making references for further promoting the construction of new green and smart cities worldwide.

## Introduction

Smart cities have the characteristics of technological innovation and green development, which helps to improve the level of green development and green total factor production efficiency of cities [1]. The construction of smart cities contributes to the green upgrading of industrial structure [2] and the transformation of consumption patterns towards conservation [3], therefore, the ecological environment quality of smart cities is higher than that of traditional cities. The environmental protection effect it possesses is one of the original intentions of China’s implementation of the smart city pilot policy. Smart cities utilize digital means to efficiently allocate resources, reduce air pollution [4], improve energy efficiency and environmental governance capabilities [5], and promote sustainable development of cities [6]. However, there are also opposing views that argue that the construction of smart cities has not reduced carbon dioxide emissions. The construction of smart cities relies on digital technology, and the application and optimization of this technology can lead to significant resource consumption, a sharp increase in carbon emissions [7], and a decrease in GTFP. There are three main reasons why the construction of smart cities instead leads to environmental pollution: consumption of large amounts of resources, pollution from the construction process, and irrational planning. Smart city construction of data centers, intelligent transportation hubs and other smart infrastructure often occupy a large amount of land and consume a large amount of resources, and the operation of these infrastructures also consumes a large amount of electricity. Especially in the process of smart infrastructure construction, construction works generate a large amount of dust and wastewater. Moreover, some smart city construction pays excessive attention to the layout of intelligent facilities, occupies a large amount of green space in order to increase intelligent facilities, and ignores ecological space planning, leading to an increase in the urban heat island effect and the risk of waterlogging. Therefore, there are two opposing views in the academic community regarding the impact of smart city construction on GTFP.

Given the existence of the two opposing views mentioned above, it is necessary to conduct a more in-depth study on the changing patterns of GTFP in the process of smart city construction. Based on the time dimension, the construction of smart cities can be divided into pre establishment, initial stage, and mid to late stage [8]. The main task during the initial establishment of a smart city is the construction of digital technology and information centers, as well as the application and promotion of digital technology. During this period, the construction of smart cities requires a significant investment in production factors and is in a stage of uneconomical production, leading to a sharp increase in carbon emissions. With the completion of these digital facilities and the popularization of information technology, not only may carbon dioxide be significantly reduced, but the built facilities also have low-carbon and carbon reduction characteristics [9]. From this, it can be seen that the GTFP may significantly decrease during the initial establishment of smart cities, but as digital facilities gradually improve, the GTFP efficiency may also correspondingly increase. From this, green and low carbon are important goals pursued in the sustainable construction of smart cities. However, existing research mainly focuses on the long-term GTFP level of smart cities, while neglecting their development changes in the early stages of construction. This may be because the main goal of early construction of smart cities is to achieve the intelligence and informatization of urban infrastructure, and policy makers may be more concerned about the technological applications and economic effects it brings, resulting in existing research mostly ignoring the problem of carbon emissions increasing significantly in the short term due to large-scale investment in digital facilities during the early stages of smart city construction. This has led to insufficient attention paid to the characteristics, reasons, and patterns of carbon emissions changes in the early stages of smart city construction, which may reduce environmental protection efforts and instead result in a surge in carbon emissions caused by smart city construction.

Based on this; to make up for this research gap, this article focuses on the GTFP in the early stage of smart city construction. Compare the changes in GTFP before, during, and after the establishment of smart cities from both dynamic and relatively static perspectives, clarify the changes in carbon emissions during the construction process, and propose scientific environmental protection policies for the early stages of smart city construction to reduce environmental damage. In addition, this article also analyzes the general patterns and individual characteristics of changes in GTFP during the initial stage of smart city establishment from both overall and individual perspectives. Considering both the general rules of overall carbon reduction in the process of smart city construction and the individual needs of carbon reduction in each city. Therefore, this study focuses on the green total factor productivity of smart cities, focusing on the initial stage of construction, comparing and measuring green total factor productivity in different time periods, applying efficiency theory to smart city analysis, expanding production efficiency theory, and enriching urban economics theory; Moreover, unifying the different stages of smart city construction, coordinating resource allocation, and maximizing resource utilization efficiency not only expands the relevant theories of urban sustainable development stages in theory, but also promotes environmental protection work in different stages of smart city construction in practice.

The research on total factor productivity of smart cities mainly focuses on three aspects. One is the impact of smart city construction on GTFP; The second is the mechanism by which this impact occurs; Thirdly, local governments are also participants in this influence mechanism, and their behavior is worth analyzing.

Firstly, some literature suggests that the construction of smart cities has improved GTFP. Smart cities aim for sustainable development [10] and solve urban problems by improving the level of green development in cities [11]. The improvement of GTFP in smart cities mainly relies on the optimization of production technology, the transformation of production processes, the reduction of harmful environmental substances, and the enhancement of environmental quality and innovation capabilities. On the one hand, enterprises in smart cities generally use digital technology to achieve enterprise management, efficiently collect data through information systems [12], optimize enterprise resource allocation based on this, and eliminate production methods with low efficiency and high pollution. Moreover, smart cities provide a platform for enterprises to communicate with each other, improving the efficiency of information communication and the speed of innovative technology exchange between enterprises, and also contributing to the improvement of green total factor efficiency [13]. On the other hand, due to the acceleration of information infrastructure construction and the expansion of communication channels between organizations in smart cities, the quality of life of residents has also been improved [14].

It can be seen that information technology plays a key role in promoting enterprise development and improving residents’ living standards in smart cities [15]. Smart cities not only accelerate the progress of green technology, but also serve as an environmental policy tool [5] that can effectively reduce environmental pollution levels through information technology [11]. The development of information technology in cities has reduced the spatial and temporal distance between cities, as well as between enterprises, and lowered the barriers to the flow of clean technology [16]. Moreover, this technology also helps to break down administrative barriers between cities, reduce communication barriers between enterprises [17], facilitate the circulation of green environmental protection technologies, and promote the improvement of urban GTFP.

Secondly, research on the mechanism of smart cities to enhance GTFP. The promotion effect of smart cities on urban GTFP has reached a consensus in the academic community, but scholars have different views on how the former affects the latter, so there are abundant research results in this area. The mechanism by which smart cities enhance GTFP mainly includes “new infrastructure”, industrial agglomeration, research and development investment, environmental protection expenditure, and digitization [18].

“New infrastructure” transforms the existing industrial system through the Internet, the Internet of Things, artificial intelligence and information technology, reshapes the industrial structure, and realizes efficient and green production [19] to alleviate industrial pollution in modern cities [20]. Therefore, the construction of smart cities represents high-quality development [21]. The “new infrastructure” project has scale effects and natural monopoly attributes in production, and green innovation features in technology [22], effectively reducing carbon emissions [23]. For example, in order to monitor the environmental protection of enterprises, the government promotes the construction and application of big data supervision systems, forcing enterprises to reduce the emission of harmful substances in the environment and improve GTFP [24]. The “new infrastructure” of smart cities not only brings about an improvement in the internal environmental quality of the city, but also generates spillover effects of green technology, driving the overall improvement of GTFP in the region [1].

Individual literature suggests that smart cities reduce green productivity through digital industrial agglomeration [25], while most of the literature suggests that GTFP increases [26]. The high penetration characteristics of digital technology [27] contribute to the improvement of urban GTFP [28]. Due to the breakthrough of digital technology beyond geographical boundaries, the matching efficiency of various production factors is higher [29]. This helps to efficiently aggregate innovative resources, leverage the agglomeration effect of digital technology, highlight the scale effect of agglomeration, highlight the spillover effect of economies of scale, and enhance the GTFP of cities and surrounding areas [30]. The agglomeration of digital industries in smart cities has brought about intensive production methods, green industrial structures, efficient production, and opened up a green path for production. Relying on the digital industry and taking the agglomeration of the digital industry as a path, we aim to enhance our technological innovation capabilities while achieving low-carbon, low-energy, and green production [31]. In addition, digital technology improves the output efficiency of various input factors by integrating into traditional industries, and also reduces energy consumption intensity, assisting in the green transformation of traditional industries [32]. The changes in residents’ lifestyles in smart cities have also led to the clustering of production methods. The digitalization and onlineization of residents’ lifestyles in smart cities, such as cloud conferencing, online shopping, and digital transactions, have rapidly become popular and achieved the same effect as offline [33]. The digital transformation of work and life has led to the online aggregation of related production factors, reducing offline energy consumption and enhancing the potential for green consumption [34]. Therefore, the transformation of production and lifestyle in smart cities has significantly reduced energy consumption and effectively improved GTFP.

Research and development investment is one of the effective ways for smart cities to enhance GTFP [35]. Smart cities can effectively enhance the innovation level of cities [36], with an increase rate of 17% -19% [37]. Due to the high investment risks in technological innovation and the external risks that may arise after successful innovation, there is a significant need for funding [38]. This leads to the need for funding for technological innovation in enterprises to assist creditors or shareholders, but they still require the enterprise to bear higher financing costs to avoid adverse selection and moral hazard [39], which may lead to technological backwardness due to a shortage of funds. To prevent enterprises from losing market competitiveness due to technological backwardness and affecting local economic development, the government may provide corresponding policy support to enterprises. Policy factors are often key to the success of technological innovation, such as industrial policies [40] and intellectual property policies [41]. Smart cities are a typical green technology innovation incentive policy [42],and the government can alleviate the burden of technological innovation on enterprises through multiple channels. One is to inject special funds into enterprise technological innovation, enhance the reputation and market share of enterprises in smart cities [43], and reduce the risk of negative externalities [44]. The second is to reduce the asymmetry of information between buyers and sellers [45]. Enterprise technical information is confidential and therefore cannot be provided to the buyer; However, the buyer cannot determine their purchasing behavior due to the inability to obtain technical information. If there is government guarantee, the cost of information authentication for both buyers and sellers will be significantly reduced. The third is preferential tax policies. In smart cities, the government’s tax incentives for technology innovation enterprises have a wide range, strong intensity, and long duration, becoming an effective incentive for enterprise technology innovation [46].

Smart cities have increased environmental protection expenditures, thereby improving GTFP. As an environmental regulation, smart cities promote the transfer of pollution intensive enterprises to other regions [47], especially to low-intensity areas [48]. For example, in the United States, companies are moving away from areas with strong environmental regulations and towards areas with looser regulations [49]. There are similar cases in Europe where EU air quality regulations force polluting companies to leave, especially those with lower green productivity that tend to lean towards areas with lower environmental costs [50]. The implementation of domestic smart city policies has also had the same effect, with polluting enterprises transferring across regions [51]. Although environmental degradation in other regions has resulted in pollution backflow effects, it is evident that smart city policies have strengthened local environmental protection and increased environmental expenditures [52]. In order to reduce environmental costs, enterprises have to complete green technology innovation [53], thereby improving their GTFP.

There is controversy over whether digitalization serves as a mediating or moderating variable for the impact of smart city policies on GTFP. Green technology is an inevitable choice for the healthy development of enterprises [54], and the digitization of smart cities provides supporting conditions for the development of green technology in enterprises [55]. Moreover, digital technology has changed the way traditional resources are obtained and allocated in smart cities [56], breaking down industry barriers and extending innovation boundaries [57]. Therefore, digital development is an intermediary variable for smart cities to enhance green comprehensive factors. Some literature suggests that digitization should be used as a moderating variable. Due to the reduction of information asymmetry in the digital economy, the integration of digital technology with various production processes enables timely disclosure of environmental information and governance capabilities of enterprises [58]. Due to the reduction of information asymmetry, the costs of various production processes in enterprises have decreased [59], enhancing the role of smart cities in promoting GTFP. Moreover, on the one hand, the reduction of information transmission barriers not only helps financial institutions evaluate and identify corporate credit, but also increases the possibility of enterprises obtaining green credit [60]. On the other hand, due to the timely feedback of information on the effectiveness of corporate environmental governance to the government, more environmental subsidies and tax incentives can be obtained. Enterprises also timely control the current situation of pollution control and develop and upgrade green technologies due to the smooth flow of production information [61]. Relying on digital technology to optimize resource allocation, produce more green products, and improve GTFP [62].

Finally, local government competition has strengthened the impact of smart city construction on GTFP [26]. Some literature suggests that the strengthening effect of local government competition has a mediating effect, while others consider it a moderating effect. The first approved smart city pilot has set an example for the green development of other cities [63]. In the “tournament” political assessment mode, local governments often increase their enforcement efforts in the process of policy implementation to win in the assessment [64]. The pilot of smart cities leverages policy advantages to obtain more high-quality resources [65], making it easier to achieve innovation in green technologies. However, other cities are at a competitive disadvantage due to not benefiting from this policy. In order to avoid being eliminated, they will learn from the development model of pilot cities and improve GTFP [63]. Therefore, competition among local governments is a mediator variable for the impact of smart city pilot policies on GTFP. Another part of the literature suggests that in the promotion “tournament” system, local governments pay more attention to the growth of the local economy [66], so innovation performance that drives significant economic growth is a key indicator that local governments focus on [67]. Smart cities promote regional digital industrialization and industrial digitization, which helps to significantly enhance innovation levels, stimulate regional economic development potential and green development momentum [68]. Therefore, digitization is a key development direction for local economies. The creation of a favorable innovation environment by local governments is an effective prerequisite for the aggregation of innovation factors, the transformation of green production methods, and the improvement of green economic efficiency [69]. Therefore, local government competition is a moderator variable for the impact of smart city governments on GTFP.

In summary, existing research has conducted extensive studies on the relationship between smart cities and GTFP. Moreover, most studies suggest that the GTFP of smart cities is on the rise. However, this result is an evaluation of the outcome of smart city construction. Since the policy orientation in the early stage of smart city construction is mainly to enhance the digital application and economic effects of the city through digital and intelligent means, this has led to less literature focusing on the changes in GTFP in the early stage of smart city construction. However, the construction of smart cities is a new infrastructure, and the input and output of factors vary in different construction periods, which may lead to different GTFP in the early, middle, and later stages of construction. With the continuous development and improvement of smart cities, policy orientation has shifted from focusing on economic effects to green economic effects. This has led most literature to focus on the middle and later stages of smart city construction, and it has been found that the GTFP of pilot cities has significantly improved after policy implementation; However, the construction of large-scale digital infrastructure is likely to bring a series of environmental pollution problems, leading to a phenomenon where the initial GTFP may decrease instead of increasing. Therefore, the characteristics of this productivity in the early stages of construction need further analysis. In view of this, the possible innovations of this article include: firstly, this article focuses on analyzing the changes in GTFP during the initial stage of smart city construction. Existing literature has not given sufficient attention to this issue, which may overlook environmental protection in the early stages of smart city construction and result in a lack of effective environmental protection strategies during this period. Secondly, this article constructs an empirical model for the characteristics of changes in GTFP during the early and middle to late stages of smart city construction. Most literature uses a double difference model to verify the effectiveness of smart city pilot policies in improving GTFP, but ignores its “U-shaped” trend, which may also lead to lower environmental protection efforts in the early stages of smart city construction. Thirdly, the biggest flaw of econometric models such as the double difference model is that they only focus on the overall average GTFP of smart cities, ignoring the characteristics of individual values. This may result in the overall development of environmental policies for smart cities not being fully suitable for individual cities, making it impossible to implement tailored policies for each city and reducing the effectiveness of environmental policies. In view of this, on the one hand, this article uses the Malmquist index and panel data model to analyze the “U” shaped change characteristics of green total factor production efficiency in the process of smart city construction as a whole, with a focus on analyzing the initial change patterns; On the other hand, using the common frontier function to analyze the characteristics of GTFP in the early stage of smart city construction at the individual level. Summarize the changes in GTFP during the initial stage of smart city construction from both macro and micro perspectives.

The research perspectives, research viewpoints, research content of the above literature and the innovations of this paper compared to the existing literature are shown in Table 1.

**Table 1:** Literature summary and contribution of this paper.

Research Perspectives	Literature	Research Contributions	research content	Contribution of this article
Relationship between smart city development and GTFP	[10–17]	The construction of smart city has improved GTFP.	This kind of research focuses on the whole process of smart city construction, and does not divide the construction stage of smart city.	This paper divides the construction of smart city into different periods.
Research on the mechanism of smart city construction to improve GTFP	[19–24]	Mechanism 1: New infrastructure	The new infrastructure realizes the high efficiency, greening and green technology spillover effect of production, and also helps the government ‘s environmental supervision.	In the early stage of smart city construction, not only did not achieve greening, but there was a decline in GTFP and an increase in pollutants.
	[25–34]	Mechanism 2: the aggregation of digital industries	Digital industry agglomeration has realized the intensification of production mode, the greening of industrial structure and the high efficiency of production. The digitization of residents ‘lifestyles leads to the online agglomeration of production factors.	
	[35–46]	Mechanism 3: R &D investment	R & D investment reduces the obstruction of green innovation caused by negative externalities and information asymmetry.	
	[47–53]	Mechanism 4: environmental protection expenditures	Smart cities have increased investment in environmental protection and improved GTFP.	
	[54–62]	Mechanism 5: digitalization	Digitization as a mediating variable or moderating variable affects GTFP.	
Local government behavior	[63–69]	Local government competition behavior strengthens the impact of smart city construction on GTFP.	The city that is first approved by the smart city has an exemplary role. Due to the competitive relationship between local governments, other cities will also increase GTFP. Local competition also helps to form a good innovation environment and further stimulate green innovation technology.	
Research method	Empirical literature	The main method of empirical literature is DID and other econometric models.	These literatures use econometric theory to analyze the impact of smart city construction on GTFP.	This paper uses Malmquist index and common frontier function model to analyze the changes of GTFP in smart cities.

## Theoretical analysis and model construction

### Theoretical analysis of the decline in GTFP in the early stage of smart city construction

Although one of the goals of smart cities is to achieve green development, there may be a decline in GTFP in the early stages of construction, mainly due to three reasons.

Reason one is that a certain amount of environmental pollutants are generated during the initial stage of smart city construction. In the initial stage of smart city construction, it is necessary to build a batch of digital infrastructure, and the construction and maintenance of facilities such as base stations, network equipment, and digital centers can cause serious environmental pollution problems [70]. In addition, carbon emissions during the operation of smart cities cannot be ignored. Take the use of the Internet as an example. For every 1% increase in Internet usage, the carbon dioxide emissions of EU countries will increase by 0.0821% [71], and OECD countries will even reach 0.16% [72]. From 1991 to 2009, the Association of Southeast Asian Nations (ASEAN) [73], especially the use of the Internet in emerging economies, led to an increase in carbon dioxide emissions [74]. This may be related to the low use density at the initial stage of Internet promotion [75], which has not reached the scale effect, and also to the low energy efficiency of information technology equipment used in these countries. The development of information technology not only promotes industrialization, but also leads to high energy consumption and an increase in carbon emissions. In the early stages of smart city construction, digital technology upgrades and the construction and operation of data centers will consume a large amount of resources, increase energy consumption, and cause environmental pollution [71].

The second reason is that in the early stages of smart city construction, there may be an energy rebound effect, leading to an increase in carbon dioxide emissions instead of a decrease. The construction and operation of smart cities are based on information technology, which may have a rebound effect in energy utilization [76]. Because the application of information technology has improved the production efficiency of enterprises, under the incentive of maximizing profits, enterprises will invest as much production factors as possible, including high carbon emissions and high pollution production factors such as energy, which leads to an increase in carbon emissions instead [77]. Moreover, due to the formation of a network economy in smart cities, the spatial spillover effect of carbon dioxide is enhanced, which also has a driving effect on the increase of carbon emissions in surrounding cities [78].

The third reason is that at the initial stage of smart city construction, due to the immaturity of digital technology, information technology, Internet technology, etc., the capacity of smart cities to manage carbon dioxide is limited [79]. In the early stage of smart city construction, the digital transformation strategy was unclear, the direction of information technology development was unclear, and there was a shortage of relevant high skilled talents. The intelligent transformation of enterprises was still in its infancy and the transformation process was slow, and the penetration rate of intelligent elements was also low [80]. Moreover, due to the low level of digital green innovation and weak innovation absorption capacity, the carbon reduction effect of smart cities cannot be effectively exerted [81]. So, in the early stages of smart city construction, carbon dioxide emissions were exacerbated. With the maturity of various technologies, the process of resource allocation is completed, and economies of scale are formed, carbon emissions can gradually decrease, and the GTFP of smart cities can be improved. For these three reasons, hypothesis 1 can be obtained.

**Hypothesis 1:** GTFP decreases at the beginning of smart city construction.

From the above reasons one and three, it can be seen that in the early stage of smart city construction, the main reason for the decrease in GTFP is that, on the one hand, the production scale is not economical and the optimal allocation of various input factors has not yet been achieved; On the other hand, factors such as immature technology and structural imbalance of labor force result in low input-output efficiency. Therefore, the reason for the decrease in GTFP in the early stage of smart city construction can be attributed to low technical efficiency. With the gradual improvement of smart city construction, the allocation of various resources is becoming more reasonable, and technological efficiency is also improving, resulting in an increase in GTFP. Therefore, most literature suggests that the GTFP of smart cities is showing an increasing trend, which is essentially a changing trend in the later stage of smart city construction.

Based on the above analysis, hypotheses 2 and 3 of this article can be derived.

**Hypothesis 2:** Technical efficiency is the main reason for changes in GTFP in smart cities.

**Hypothesis 3:** After the implementation of the smart city pilot policy, the GTFP of the pilot cities shows a trend of first decreasing and then increasing.

**Fig 1:**
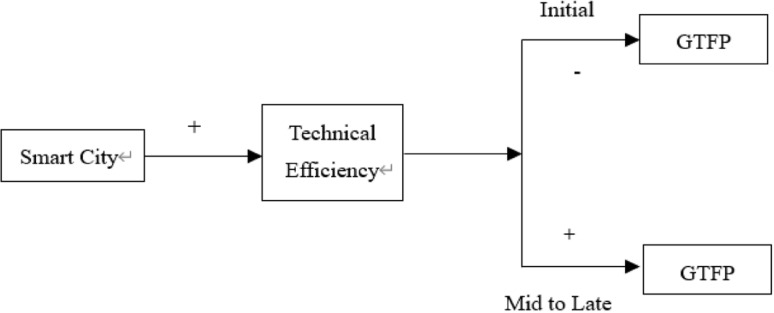
Theoretical idea.

The theoretical idea is shown in Fig 1.

### Model construction of the impact of smart city construction on GTFP

Construct a panel data model, Malmquist index, and common frontier function to verify the theoretical hypotheses proposed in this paper from both the overall and individual perspectives of smart cities.

#### Malmquist index.

The main methods for measuring and analyzing the frontier of GTFP include the Sto-chastic Frontier Approach (*SFA*), the non-parametric method (*Data Envelopment Analysis, DEA*), the Slack- Based-Measure (*SBM*), and Malmquist Index. Parametric methods require the measurement of efficiency under the assumption of a frontier function. The non-parametric method does not need to assume the frontier function in the performance calculation process, and more often uses data envelopment analysis for measurement. DEA has an advantage over SFA because multiple input and output indicators are considered when measuring GTFP in smart cities. However, the DEA model cannot consider the impact of adverse environmental outputs and thus cannot measure the real level of GTFP, while the SBM can effectively solve the above problem. However, the SBM model also has some limitations, which can only measure the relative efficiency of the cross-section pilots, but cannot effectively observe the trend of GTFP in smart cities, and the Malmquist index can effectively make up for the shortcomings of the above methods, which can not only take into account the non-desired outputs brought by the construction of the smart city and more accurately calculate the level of GTFP of the smart city, but also effectively observe its dynamic trend and explore the trend of GTFP in smart cities. It can also effectively observe the trend of its dynamic changes and explore its causes. And, according to the theoretical analysis of this paper, the GTFP may decline in the early stage of smart city construction and may be enhanced in the middle and late stages, which is a dynamic change process that first declines and then rises, and this dynamic change is suitable for index analysis. Therefore, this paper applies to the Malmquist Index to analyze the trends and causes of GTFP in smart cities.

The Malmquist index is shown in equation (1). Equation (1) adopts geometric mean solution to avoid the problem of inconsistent calculation results due to different time periods. *MI* represents the *Malmquist* index, and (*t*, *t* + 1) represents the period from t to t + 1. Using the *t*-th period as the technical reference plane, represent the input-output volume of the *t*-th period. If MI>1 , it indicates an increase in GTFP of smart cities; If 0<MI<1, it indicates a decrease in production rate. Furthermore, equation (1) can be transformed into equation (2), which decomposes into two equations, namely the technological progress index and the technological efficiency index, as shown in equations (3) and (4), respectively. If TECH>1, it indicates that technological progress is the reason for the increase in GTFP; On the contrary, there has been a relative technological decline and a decrease in GTFP. If EFFCH>1, it indicates that the improvement in technical efficiency is the reason for the increase in GTFP; On the contrary, it indicates a decrease in technological efficiency and a decrease in GTFP.


$$\begin{array}{c} MI(t,t+1)={\left[\frac{D^{t}(K^{t+1},L^{t+1},A^{t+1},Y^{t+1},C^{t+1})\cdot D^{t+1}(K^{t+1},L^{t+1},A^{t+1},Y^{t+1},C^{t+1})}{D^{t}(K^{t},L^{t},A^{t},Y^{t},C^{t})\cdot D^{t+1}(K^{t},L^{t},A^{t},Y^{t},C^{t})}\right]}^{\frac{1}{2}} \end{array}$$ (1)

$$\begin{array}{c} MI(t,t+1)=\frac{D^{t}(K^{t+1},L^{t+1},A^{t+1},Y^{t+1},C^{t+1})}{D^{t+1}(K^{t},L^{t},A^{t},Y^{t},C^{t})}{\left[\frac{D^{t+1}(K^{t+1},L^{t+1},A^{t+1},Y^{t+1},C^{t+1})\cdot D^{t+1}(K^{t},L^{t},A^{t},Y^{t},C^{t})}{D^{t}(K^{t},L^{t},A^{t},Y^{t},C^{t})\cdot D^{t}(K^{t+1},L^{t+1},A^{t+1},Y^{t+1},C^{t+1})}\right]}^{\frac{1}{2}} \end{array}$$ (2)

$$\begin{array}{c} TECH={\left[\frac{D^{t+1}(K^{t+1},L^{t+1},A^{t+1},Y^{t+1},C^{t+1})\cdot D^{t+1}(K^{t},L^{t},A^{t},Y^{t},C^{t})}{D^{t}(K^{t},L^{t},A^{t},Y^{t},C^{t})\cdot D^{t}(K^{t+1},L^{t+1},A^{t+1},Y^{t+1},C^{t+1})}\right]}^{\frac{1}{2}} \end{array}$$ (3)

$$\begin{array}{c} EFFCH=\frac{D^{t}(K^{t+1},L^{t+1},A^{t+1},Y^{t+1},C^{t+1})}{D^{t+1}(K^{t},L^{t},A^{t},Y^{t},C^{t})} \end{array}$$ (4)

#### Panel data model.

Although the Malmquist index not only has the ability to analyze the trend of changes in GTFP, but also can trace this change back to technological progress and efficiency, it cannot identify concrete influencing factors, which is not conducive to the improvement of GTFP. For example, based on the theoretical analysis in this article, it can be concluded that technical efficiency is the main factor affecting GTFP. In the construction of smart cities, technological efficiency mainly refers to the degree to which the potential of information technology and digital technology is fully utilized. However, the technical efficiency in the Malmquist index is relatively abstract and cannot be linked to specific technological input factors in smart city construction, thus unable to determine the key technological factors for improving GTFP in smart cities. Therefore, this article constructs a panel data model to determine the concrete indicators of technical efficiency on the one hand, and further verify that technical efficiency is the main influencing factor of GTFP in smart cities, and this influence shows a U-shaped change characteristic of first decreasing and then increasing.

The panel data model constructed in this article is specifically shown in equation (5). The GTFP (*Y*) of smart cities is the dependent variable, and the technical efficiency (*X*) of smart cities is the core independent variable, combined with the control variable (*Control*) to form a panel data model.


$$Y_{it}=\beta_{0}+\beta_{1}X_{it}^{2}+\beta_{2}X_{it}+\beta_{3}Control_{it}+\varepsilon_{ict}$$ (5)

#### Common frontier function.

The Malmquist index and panel data model validated the average trend and influencing factors of GTFP changes in smart city construction. Moreover, both models can also verify whether this trend of change is caused by changes in technical efficiency. However, the Malmquist index focuses on analyzing the dynamic changes in GTFP and cannot determine whether the GTFP of individuals in smart cities has reached a relatively optimal level. Therefore, it is necessary to analyze the GTFP of individuals in smart cities through relative static comparisons, and explore their improvement paths based on this, to guide targeted carbon reduction in the early stages of smart city construction.

**Fig 2:**
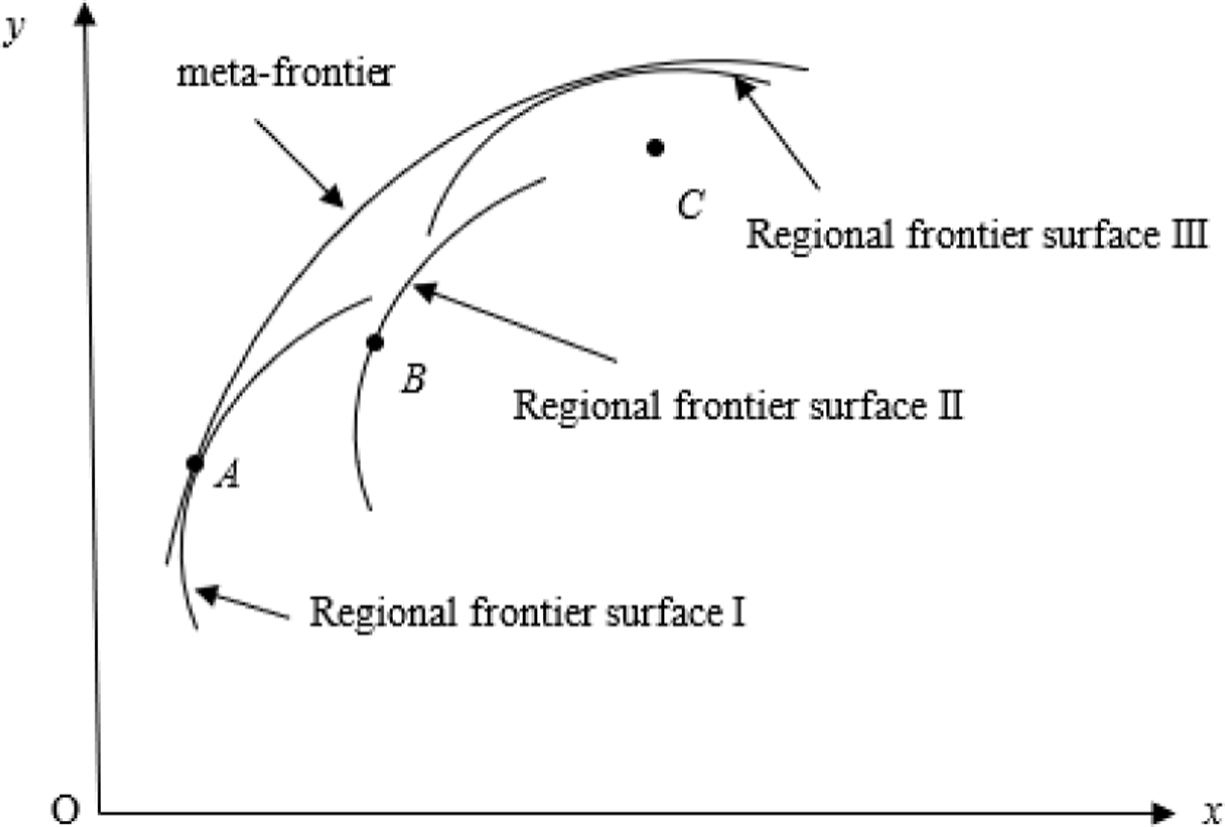
Meta-frontier and regional frontier.

The construction method of the common frontier function is shown in Fig 2. Assuming there are three regions, namely Region I, Region II, and Region III, each region inputs a certain amount of production factors and produces multiple outputs, including at least one undesirable output, such as carbon dioxide, whose production process is $P^{k}(x)=\left\{(y,c):(x,y,cinT^{k}\right\}$. $P^{k}(x)$ is the production set, which constitutes the production front or regional front, y is the desirable output, c is the undesirable output, and *x* is the production factor. The three production sets in the three regions in Fig 1 form the front faces of the three regions. If a city’s production is located at the forefront of the region, it means that compared to other cities in the region, the city’s GTFP is the highest in the region. If a city is not at the forefront of the region, it means that it has not reached the maximum value of GTFP in the region. The gap between urban and regional frontiers can be calculated using the *Shephard distance function*, i.e., $D^{k}(x,y,c)=\sup\left\{\lambda :(x,y,\frac{c}{\lambda }inP^{k}(x)\right\}$. λ is the maximum reduction ratio of carbon dioxide in the sample city relative to the frontier of the region. The larger this ratio, the lower the GTFP of the sample city compared to the city with the best GTFP in the region.

The regional production frontier constitutes a meta-frontier. The meta-frontier forms a frontier function output set $P^{*}(x)$ by enveloping the production frontiers of multiple regions, and its distance function is $D^{*}(x,y,c)=\sup\left\{\lambda :(x,y,\frac{c}{\lambda }inP^{*}(x)\right\}$. As shown in Fig 2, the regional frontier of the three regions forms a meta-frontier. Among them, the regional frontier I and III are on the Meta-frontier, while the regional frontier II is not on the Meta-frontier. Points A, B, and C belong to the regional frontier I, II, and III, respectively. Point A is located on both the meta-frontier and the regional frontier; Point B is on the regional frontier, but not on the meta-frontier, and point C is neither on the regional frontier nor on the meta-frontier. This means that whether using the regional frontier I or the meta-frontier as a reference surface, the GTFP of sample city A reaches its maximum, i.e., $D^{A}(x,y,ctext=D^{*}(x,y,c)$; In Region II, the GTFP of sample city B reached the maximum value in the region, but did not reach the maximum value referenced by the meta-frontier, that is $D^{B}(x,y,c)<D^{*}(x,y,c)$; By comparison, the GTFP of sample city C in region III not only has room for improvement based on the regional frontier, but also has not reached its maximum value when using the meta-frontier as a reference surface. From this, the technology gap ratio (TGR) under meta-frontier and regional frontier can be obtained, $TGR^{k}(x,y,c)=\frac{D^{k}(x,y,c)}{D^{*}(x,y,c)}$. The larger the TGR, the greater the gap between the regional frontier and the meta-frontier, that is, compared with the region at or near the meta-frontier, the GTFP of the region is lower.

The calculation of the distance function is shown in equation (6).


$$\mathrm{\backslash [}\begin{array}{c} \begin{array}{c} \left[D(x_{i},y_{i},c_{i})\right]=\text{min}\;\rho \\ s.t.\sum_{i=1}^{k}\lambda_{i}x_{i}\leq x_{i};\sum_{i=1}^{k}\lambda_{i}y_{i}\geq y_{i};\sum_{i=1}^{k}\lambda_{i}c_{i}=\rho c_{i};\lambda_{i}\geq 0;i=1,2,\cdots ,k \end{array} \end{array}\mathrm{\backslash ]}$$ (6)

## Empirical analysis

### Selection of samples and indicators

#### Sample selection.

The Ministry of Housing and Urban Rural Development announced the first batch of national smart city pilot list on January 29, 2013. The total number of pilot cities in this list is 90, including 37 prefecture level cities, 50 districts/counties, and 3 towns; On August 5, 2013, the second batch of national smart city pilot list was announced. This list includes 103 sample regions, including both cities and districts; On April 7, 2014, the third batch of national smart city pilot list was announced, with a total of 84 sample areas, including both newly added pilot areas and the expansion of the scope of the first and second batches of pilot areas.

The list of pilot cities includes both cities and counties or districts. In order to ensure consistency in statistical standards, this article selects cities as samples. If only some districts or counties in the city are pilot areas, they are not within the scope of this study. Based on this, the first batch of smart city pilot projects includes 37 cities, the second batch includes 42, and the third batch includes 31. (Smart City Pilot Policy cities include:: Shijiazhuang, Qinhuangdao, Langfang, Handan,et al. Due to space constraints, this paper does not list all three batches of smart cities, if you need to contact the corresponding authors).The method for determining the investigation time is as follows. The first batch of smart city construction began in early 2013, making it the first year of its construction. The second and third batches of smart city construction began in August 2013 and April 2014 respectively, and by the end of that year, they had not been completed for a year. Therefore, the implementation of these two batches of smart city pilot construction was postponed by one year, to 2014 and 2015 respectively. In order to compare with the implementation effect of smart city pilot construction, a total of 60 cities in various provinces and regions of China that have not implemented smart city pilot construction were selected using stratified sampling method to form a control group (The control group cities include Chengde, Hengshui, Jinzhong, Linfen,et al.. Due to space constraints, this paper does not list all 60 cities in the control group, if you need to contact the corresponding authors) During the sampling process, each province should be selected, and cities should be selected using the ratio of equality method in stratified sampling method.

The survey period corresponding to the panel data model is 2011–2021, and three batches of smart city pilots are selected as research objects. The reason for this choice is that although 2013 was the first batch of smart city pilot policies to be implemented, compared with before the policy was implemented, the research time was advanced to 2011.

#### Selection of indicators.

Explained variable

GTFP (*Mal*) is the explained variable of this paper, which is calculated based on the common frontier function and Malmquist index model. Based on the full consideration of existing research, this paper constructs the GTFP evaluation index system of smart cities by comprehensively considering the scientific and accessibility of the indexes, as shown in Table 2.

**Table 2:** Indicator system.

Variable	Specific Indicator	Indicator Description	Unit
Input variable	Capital stock	Fixed capital	billion yuan
	Labor	Annual average number of employees	10,000 people
	Energy consumption	Total energy consumption	million tonnes
Output variable	Desired output	Real GDP	billion yuan
	Undesired output	Carbon dioxide emissions	million tonnes

(1) Selection of Input Indicators

With reference to existing studies [82], this paper selects capital, labor and energy as input indicators. Capital input is measured by regional fixed capital stock, which is converted to 2011 prices using the perpetual inventory method. Labor input is measured by the annual average, which is the arithmetic means of the number of people in the labor force at the beginning and end of the year. Energy consumption is converted to standard coal by converting the city’s electricity, natural gas, gas and liquefied petroleum gas usage. As this paper studies GTFP, both capital inputs and labor inputs related to smart city production are input factors, and no distinction is made between smart and non-smart input factors; the same treatment is adopted for outputs.

(2) Selection of output indicators

Since GTFP is a comprehensive indicator that includes environmental and economic benefits, this paper selects output indicators from both desired output and non-desired output. Specifically, the desired output mainly refers to the economic benefits, and this paper selects the total real GDP to measure; while the non-desired output mainly refers to the environmental pollution produced in the process of urban development, and this paper selects the carbon dioxide emissions to measure. Carbon dioxide in the city is measured by adding the amount of carbon dioxide produced by electricity, gas and liquefied petroleum gas (*LPG*), and thermal energy consumption. The amount of carbon dioxide produced by gas and LPG is calculated using the conversion factors in IPCC2006, the carbon dioxide produced by electricity is calculated based on the baseline emission factor of the grid and the amount of electricity consumed, and the carbon dioxide produced by heat is calculated based on the consumption of raw coal [83].

Explanatory variables

The explanatory variable in this paper is technical efficiency, i.e., the application of innovative technologies. The key technologies in the construction of smart cities are digital and information technologies, and the application of these technologies, i.e., the process of investing in digital infrastructure, can be described by this indicator in terms of information technology and communication productive capital (*ITC*) [84].

Control variables:

This paper combines the existing research and the actual situation, and selects the following variables as the control variables of this paper:

(1) The level of economic development (*GDP*): on the one hand, an increase in output leads to the more pollution generated by economic activities; on the other hand, the increase in the level of economic development constantly puts forward new requirements for the green development of cities [74].Therefore, the level of economic development may be an important factor influencing the level of GTFP in smart cities. In this paper, the constant price per capita GDP with 2003 as the base period is selected as an indicator of the city’s economic development level.
(2) Openness to the outside world (*Open*): on the one hand, regions with a higher degree of openness to the outside world will have easier access to advanced green technologies and management experience from abroad, thus enhancing the GTFP of the region; on the other hand, some regions may choose to relax the standards of environmental regulations in order to introduce the entry of foreign-funded enterprises, thus introducing some high-energy-consuming and high-polluting enterprises, which will impede the enhancement of the region’s GTFP [76]. On the other hand, some regions may choose to relax environmental regulation standards to introduce foreign enterprises, thus introducing some high energy-consuming and highly polluting enterprises, hindering the improvement of the region’s GTFP. Therefore, the degree of opening to the outside world is an important factor affecting the level of regional GTFP. In this paper, the ratio of the amount of foreign capital used by foreigners to GDP is chosen to measure the degree of openness of the city.
(3) City size (*Scal*). On the one hand, large-scale cities tend to attract more talents, technology, capital and other factors of production, and the agglomeration of these factors can enhance the efficiency of resource allocation, promote the synergistic development of different industries, and promote the development of high-end industries, thus enhancing the GTFP of the region; on the other hand, large-scale cities are more densely populated and economically active, which may result in the shortage of resources, environmental pollution, and other problems, thereby On the other hand, large-scale cities with dense population and economic activities may cause resource shortage, environmental pollution and other problems, thus hindering the improvement of GTFP [79]. Therefore, city size is an important factor affecting GTFP. In this paper, the ratio of urban resident population to GDP is chosen as a measurement indicator.
(4) Environmental regulation intensity (*EnvRe*). Regions with stronger environmental regulations have relatively less pollution emission and their GTFP level is relatively high. However, excessive environmental regulation will, to a certain extent, harm the economic efficiency of the region, which is not conducive to the enhancement of the level of GTFP [80]. Therefore, this paper selects the inverse of industrial smoke (dust) emissions per unit of GDP to measure the intensity of urban environmental regulation as one of the control variables in this paper.
(5) Infrastructure level (*Inf*). The improvement of regional infrastructure level can not only bring about the improvement of resource allocation efficiency level but also drive the optimization and upgrading of industrial structure by accelerating the diffusion of technology, information spillover, etc., which can help to enhance the GTFP level of the region. Therefore, this paper selects the level of infrastructure as one of the control variables and adopts the length of urban roads per capita as a measurement indicator.

#### Data sources.

Fixed assets investment, employment, GDP and other social and economic data are mainly from China Urban Statistical Yearbook and China Statistical Yearbook over the years. The data for measuring carbon dioxide emissions, such as energy consumption, mainly comes from the China Environmental Statistical Yearbook. The data for measuring technological efficiency in information technology and communication productive capital comes from the “China Electronic Information Industry Yearbook”. All four statistical yearbooks are available from the China Economic and Social Big Data platform. To further enhance the scientific validity of the data, this article excluded research samples with severe missing key data. Individual missing data are supplemented by interpolation or the average of the previous and subsequent years. In addition, all nominal prices are converted to actual prices priced in 2011.

### Empirical results and discussion

#### Descriptive statistics.

The results of descriptive statistics of relevant variables are shown in Table 3. The maximum value of GTFP is 2.141, the minimum value is 0.103, and the standard deviation is 2.432, which indicates that there is a large gap in the level of GTFP among the sample cities. The maximum value of information technology investment is 377,867.1 billion yuan, the minimum value is 0.143 billion yuan, and the standard deviation is 420.335, which indicates that there is a large gap in the level of information technology investment among the sample cities. Other variables are like the descriptive statistics of existing studies and will not be repeated here.

**Table 3:** Descriptive statistics.

Variable	unit	sample size	Min value	Max value	mean	STD
*Mal*	/	259	0.103	2.141	1.103	2.432
*ITC*	billion yuan	259	0.143	3778,671	210.532	420.335
*GDP*	million yuan	259	1.567	3.800	2.491	56.782
*Open*	/	259	0.021	0.403	0.202	0.781
*Scal*	million people	259	3.725	8.052	5.201	1.332
*EnvRe*	/	259	0.052	0.417	0.133	0.503
*Inf*	kilometer	259	2.053	6.141	3.722	4.021

#### Trend and index decomposition analysis of GTFP index in smart cities.

**Fig 3:**
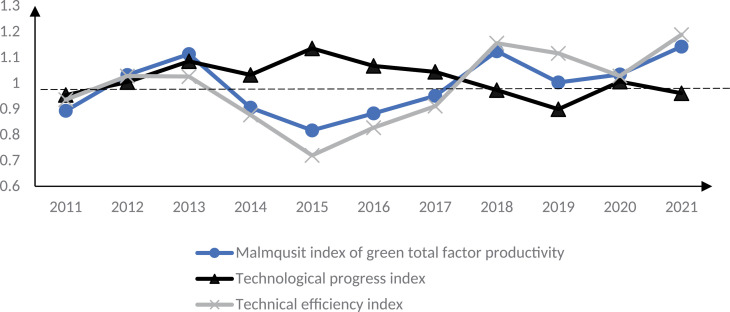
GTFP of the first batch of smart cities in 2011–2021.

**Fig 4:**
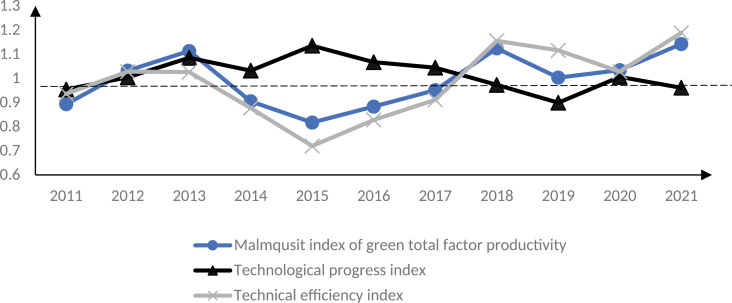
GTFP of the second batch of smart cities in 2011–2021.

**Fig 5:**
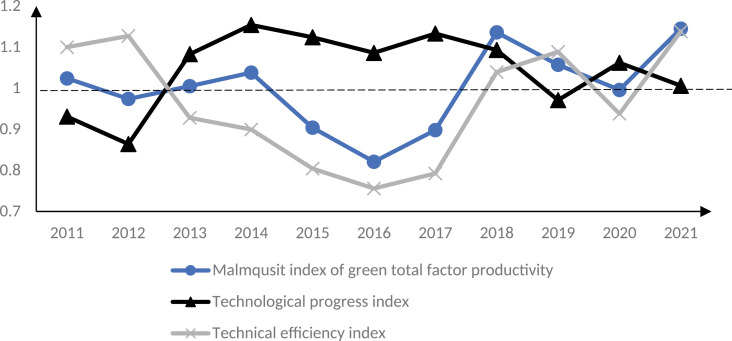
GTFP of the third batch of smart cities in 2011–2021.

**Fig 6:**
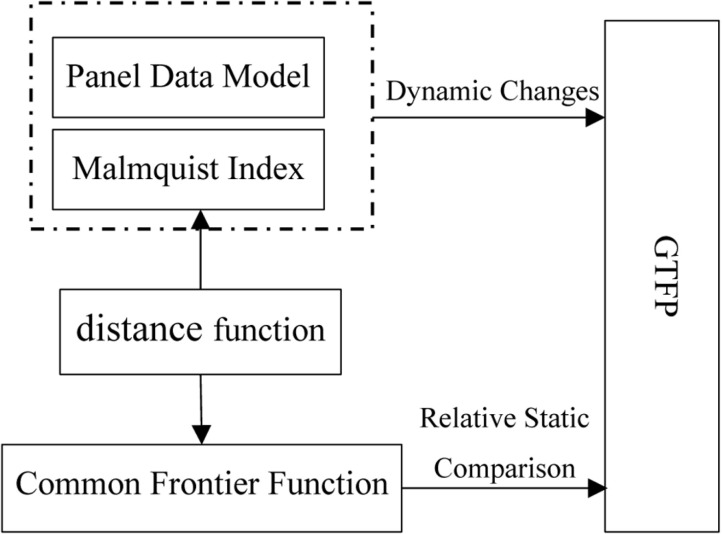
Methodological relationship.

Use equation (1) Malmquist index to verify the theoretical analysis results of this paper. To highlight the trend of changes in GTFP in smart cities and reduce the interference of sample individuals, the time series of the average value of Malmquist index of smart city construction and control group are analyzed, as shown in Figs 3–5. 2013 is the first year of the first batch of smart city construction, as shown in Fig 3. Before 2013, i.e., before the implementation of the smart city pilot policy, the Malmquist index of GTFP of the smart city pilot was greater than 1. In the early stage of the policy implementation, i.e., 2013–2015, this index was less than 1, and the Malmquist index of GTFP of the smart city in 2014 was around 0.7, which was at the three years’ lowest level. This indicates that the GTFP of the first batch of smart city pilots showed a decreasing trend in the early stage of policy implementation. The reason for the above phenomenon may be due to the need to build and maintain digital infrastructure such as base stations, network equipment, digital centers, etc. at the initial stage of smart city construction, which may lead to more serious environmental pollution problems. Moreover, the lack of digital technology penetration and the low level of digital green innovation in the early stage of the construction of smart cities cannot effectively reduce carbon emissions and other environmental pollution problems, resulting in a downward trend in their GTFP, which verifies the hypothesis of this paper.1 As can be seen in Fig 6, this trend was reversed in 2015, and the GTFP of the first batch of smart cities was close to 1 in 2015, and from 2016 the GTFP is greater than 1 in most years from 2016, i.e., the GTFP increases. This indicates that the green TFP of smart cities decreases in the short term, while in the medium and long term it shows an increasing trend. The reason for this may be that as the construction of smart cities advances, the infrastructure continues to be improved, information technology and digital technology continue to be used, and the quality of the environment improves, the GTFP also increases. At the same time, some smart cities have formulated policies to encourage the development of green industries in the middle and late stages, such as financial subsidies, tax incentives and credit support for environmental protection, new energy and resource recycling industries, etc.; and through the establishment of a special fund for green technological innovation and the elimination of standards for high-energy-consuming industries, enterprises are encouraged to carry out green innovation activities and reduce environmental pollution, which has effectively increased the GTFP of smart cities. GTFP level of the smart city. Therefore, in the process of smart city construction, GTFP shows a trend of decreasing first and then increasing, which verifies the hypothesis 3 of this paper.

The Malmquist index of GTFP for the second and third batches of smart cities shows similar results. In Fig 4, the second batch of smart city pilot policies started to be implemented in 2014, and until 2017, the GTFP Malmquist index of this batch of pilot cities is less than 1, and it is reduced to about 0.7 in 2015, and this index is greater than 1 only from 2018. This suggests that the second batch of smart city pilots’ GTFP decreases at the early stage of the policy implementation and increases in the middle and late stages of policy implementation, further proving Hypotheses 1 and 3 of this paper. Similar results also appear in the construction process of the third batch of smart city pilots, as shown in Fig 5. Before the construction of the third batch of smart cities, the GTFP Malmquist index of the pilot cities fluctuated in the 1 annex from 2011 to 2014; in the first three years after the launch of the smart city construction, i.e., from 2015 to 2017, this index was less than 1. After 2018, this index was again greater than 1. Figs 3–5 supplement 6 validate Hypotheses 1 and 3. The above results are in line with Wang et al. [11] and Zhang et al. [12], who concluded that smart cities reduce environmental pollution and enhance the level of GTFP on the whole, whereas the study in this paper found that the initial period of smart city implementation would have a negative impact on the enhancement of GTFP. According to the results of the study, it can also be found that although the initial digitalization and informatization construction will produce certain environmental pollution problems in the short term, resulting in a reduction in the level of GTFP; however, from the point of view of the city’s long-term sustainable development, the digitalization infrastructure construction will lead to the transformation and upgrading of the regional industrial structure, and the enhancement of the level of green technological innovation, which will help to increase the GTFP of the city. Therefore, from the perspective of sustainable economic development, the short-term reduction of GTFP in smart cities is not entirely negative. From this, it can be seen that when implementing smart city policies in other countries, low-carbon building materials and renewable energy technologies should be prioritized in the early stages of smart city construction, and the application of energy-saving technologies should be actively promoted; At the same time, in the early stage of smart city construction, dynamic monitoring systems can be installed next to large infrastructure to monitor pollution emissions, energy consumption, and other issues during construction, in order to reduce environmental pollution such as carbon emissions in the early stage of smart city construction and improve the GTFP level in this stage.

The GTFP Malmquist index is decomposed into equations (3) and (4), which are the technical progress index and technical efficiency index, respectively. From Figs 3–5, it can be seen that the change trend of the technical efficiency index of the smart city pilot is consistent with that of the GTFP Malmquist index, which verifies Hypothesis 2, i.e., changes in technical efficiency are the main reason for the changes in the GTFP, compared with the limited impact of the technical progress index on the GTFP index, and even the trend of the two changes is not consistent. This indicates that technological progress does not directly affect the output capacity of enterprises. This is because the impact of technological progress on GTFP may require three steps. First, new technology can be transformed into real productivity, i.e., technological progress can be applied in actual production; second, technological progress improves technological efficiency. Although the pilot cities achieved digital technological progress at the early stage of smart city construction, the technological efficiency is low because it is at the early stage of technological progress, which has not yet come into play, and the allocation of factors of production has not yet been adjusted to the optimal structure; finally, technological efficiency enhancement promotes the growth of GTFP. As the scope of information technology application to production continues to expand, the green production capacity of enterprises is also gradually enhanced, and GTFP increases. The above findings are consistent with those of Liu et al. [23] and Luo et al. [26], whose studies found that technical efficiency is a key influence on GTFP in smart cities.

The results of the study confirm the three research hypotheses of this paper. The above findings are mainly because many digital infrastructures need to be built at the early stage of smart city construction, and the start-up, operation and maintenance of these facilities require large-scale resource consumption and generate a huge amount of carbon dioxide. Because of the early stage of smart city construction, the input and use of resources do not reach the scale effect, and the allocation of factors in various production areas is also inefficient, so the GTFP is reduced. Moreover, although the GTFP is low at the early stage of smart city construction, the production efficiency increases due to the wide application of digital technology and information technology. Under the goal of profit maximization, enterprises increase the input of production factors such as energy to create more revenue while increasing the emission of carbon dioxide. Moreover, due to the backwardness of digital and information technology in this period, no effective carbon reduction technology has been formed, which also leads to the reduction of GTFP in the early stage of smart city construction. Therefore, the GTFP decreases in the early stage of smart city construction. However, as the construction of the smart city advances, the infrastructure continues to improve, and information technology and digital technology continue to be used, the environmental quality improves, and the GTFP increases. Moreover, to effectively solve the problem of increased initial pollution, emissions brought about by smart cities, governments at all levels have introduced policies such as special green technology funds, tax incentives, R&D subsidies, etc., which have effectively improved technological efficiency and thus increased GTFP. Therefore, during the construction of smart cities, GTFP shows a trend of decreasing first and then increasing, and the change of technical efficiency is the key reason for the above trend of change. From this, it can be seen that when promoting smart city policies in other countries, in the early stages of smart city construction, comprehensive deployment of IoT sensing devices, including sensors, smart meters, etc., can be carried out to achieve real-time data collection and monitoring of urban environment, infrastructure, public services, and other aspects, providing a rich data foundation for the operation of smart cities and thereby improving the technical efficiency level of this stage; At the same time, other countries can integrate data resources from various departments and fields of the city in the early stages of smart city construction by building a unified data sharing platform, breaking down data silos, achieving centralized management and sharing exchange of data, thereby improving technical efficiency in the early stages of construction and achieving an increase in the level of GTFP in the city. In the later stage of the construction of smart cities, the coverage of IoT technology in various fields of the city can be further expanded to achieve wider device interconnection and data collection. In industrial production, remote monitoring and intelligent operation and maintenance of equipment can be achieved through IoT technology, improving production efficiency and reducing energy consumption. Through the above methods, further improve technical efficiency and achieve overall improvement in urban GTFP.

This paper enriches the theory of sustainable urban development stages and strengthens the understanding of technology-driven sustainable urban development. While traditional theories of sustainable urban development emphasize a continuous and gradual development model, the GTFP change model of smart cities suggests that there may be stages of sustainable urban development, and that changes in technological efficiency are a key factor in this trend. While the initial investment and adjustment of large amounts of digital infrastructure may result in a certain ‘pain period’ of green development, smart cities will enter a more efficient and sustainable phase of development as the technology and management model matures. From this, other countries should pay attention to the phased characteristics of urban sustainable development caused by changes in technological efficiency brought about by digitization and informatization when implementing smart city policies. In the early stages of smart city construction, considering the potential environmental pollution caused by large-scale infrastructure construction, relevant departments at all levels can minimize pollution emissions by using green and low-carbon building materials, clean energy technologies, and other methods; In the middle and later stages of smart city construction, policies related to research and development subsidies, tax incentives, etc. can be formulated and implemented through the establishment of data centers, talent exchange platforms, etc., to further improve the technical efficiency of enterprises and achieve the overall improvement of GTFP in smart cities.

#### Panel data analysis of factors influencing GTFP in smart cities.

Although the Malmquist index decomposition analysis shows that the reason for changes in GTFP in smart cities is technical efficiency, technical efficiency is a relative indicator rather than an absolute indicator. This leads to the lack of concrete technical efficiency indicators, which is not conducive to formulating feasible plans for improving GTFP in smart cities. Therefore, this article takes the first, second, and third batches of smart city pilot as the research objects, with Malmquist index as the dependent variable and information technology and communication productive capital (*ITC*) as the core independent variables, combined with relevant control variables, according to equation (5), the relevant results of *ITC’*s impact on GTFP can be obtained, as shown in column (1) of Tables 4–6. To verify the robustness of the panel data model, the following four methods are adopted. Firstly, Green patent applications instead of Malmquist index as dependent variable. Secondly, Delete the smallest 5% of data and the largest 5% of data in the sample. Thirdly, Weak endogeneity samples are eliminated. Municipalities and provincial capitals are removed to avoid disturbances caused by unbalanced characteristics of cities of different sizes. Fourthly, Data for 2017–2019 was deleted to avoid the impact of encouraging application for a smart city in those three years. The results of the robustness test are shown in columns (2) - (5) of Tables 4–6.

**Table 4:** The regression results and robustness test of the first batch of smart cities.

Variable	(1)	(2)	(3)	(4)	(5)
*ITC*	1.123^**^ (0.562)	1.610^***^ (0.442)	1.421^***^ (0.892)	1.228^***^ (0.685)	1.226^***^ (0.794)
*ITC* ^2^	0.473^***^	0.571^***^	0.556^**^	0.482^**^	0.453^**^
	(0.071)	(0.301)	(0.190)	(0.025)	(0.571)
*GDP*	1.303^*^	1.212^**^	1.262^***^	1.373^**^	1.261^*^
	(0.796)	(0.841)	(0.833)	(0.304)	(0.593)
*Open*	0.701^**^	0.832^**^	0.843^***^	0.742^***^	0.863^**^
	(0.703)	(0.691)	(0.586)	(0.490)	(0.665)
*Scal*	0.827^***^	0.779^***^	0.635^**^	0.723^***^	0.773^***^
	(0.551)	(0.433)	(0.242)	(0.504)	(0.142)
*EnvRe*	0.485^**^	0.625^**^	0.595^*^	0.447^**^	0.579^**^
	(0.068)	(0.303)	(0.084)	(0.501)	(0.485)
*Inf*	0.045^*^	0.104^***^	0.114^*^	0.105^***^	0.077^*^
	(0.035)	(0.807)	(0.051)	(0.069)	(0.071)
Constant	1.551^**^	1.632^***^	1.297^**^	1.473^***^	1.553^***^
	(0.402)	(0.519)	(0.384)	(0.541)	(0.692)
District effects	Control	Control	Control	Control	Control
Time effect	Control	Control	Control	Control	Control
Sample size	259	259	259	259	259
*R* ^*2*^	0.604	0.551	0.662	0.582	0.473

***, ** and * represent significant levels of 1%, 5% and 10%, respectively.

Data source: calculated.

**Table 5:** The regression results and robustness test of the second batch of smart cities.

Variable	(1)	(2)	(3)	(4)	(5)
*ITC*	1.231^**^ (0.427)	1.355^***^ (0.173)	1.277^***^ (0.470)	1.324^***^ (0.302)	1.307^***^ (0.312)
*ITC* ^2^	0.296^***^	0.441^***^	0.368^**^	0.351^**^	0.317^**^
	(0.085)	(0.255)	(0.087)	(0.128)	(0.078)
*GDP*	1.518^*^	1.305^**^	1.368^***^	1.252^**^	1.026^*^
	(0.661)	(0.792)	(0.533)	(0.449)	(0.337)
*Open*	0.845^**^	0.881^**^	0.735^***^	0.805^***^	0.792^**^
	(0.203)	(0.304)	(0.214)	(0.197)	(0.203)
*Scal*	0.772^***^	0.638^***^	0.758^**^	0.681^***^	0.662^***^
	(0.491)	(0.351)	(0.192)	(0.331)	(0.085)
*EnvRe*	0.372^**^	0.418^**^	0.443^*^	0.352^**^	0.392^**^
	(0.068)	(0.086)	(0.076)	(0.075)	(0.062)
*Inf*	0.071^*^	0.126^***^	0.095^*^	0.083^***^	0.089^*^
	(0.033)	(0.064)	(0.049)	(0.036)	(0.041)
Constant	2.411^**^	1.883^***^	1.903^**^	1.706^***^	1.631^***^
	(0.380)	(0.203)	(0.098)	(0.128)	(0.127)
District effects	Control	Control	Control	Control	Control
Time effect	Control	Control	Control	Control	Control
Sample size	286	286	286	286	286
*R* ^*2*^	0.582	0.603	0.622	0.591	0.638

***, ** and * represent significant levels of 1%, 5% and 10%, respectively.

Data source: calculated.

**Table 6:** The regression results and robustness test of the third batch of smart cities.

Variable	(1)	(2)	(3)	(4)	(5)
*ITC*	1.058^**^ (0.228)	1.241^***^ (0.082)	1.152^***^ (0.070)	1.215^***^ (0.421)	1.163^***^ (0.228)
*ITC* ^2^	0.157^***^	0.315^***^	0.247^**^	0.268^**^	0.318^**^
	(0.078)	(0.103)	(0.092)	(0.083)	(0.132)
*GDP*	1.481^*^	1.261^**^	1.261^***^	1.328^**^	1.326^*^
	(0.452)	(0.321)	(0.434)	(0.393)	(0.207)
*Open*	0.608^**^	0.716^**^	0.616^***^	0.726^***^	0.695^**^
	(0.172)	(0.216)	(0.147)	(0.261)	(0.176)
*Scal*	0.596^***^	0.588^***^	0.637^**^	0.552^***^	0.537^***^
	(0.153)	(0.073)	(0.125)	(0.078)	(0.113)
*EnvRe*	0.572^**^	0.551^**^	0.617^*^	0.627^**^	0.624^**^
	(0.108)	(0.261)	(0.142)	(0.104)	(0.128)
*Inf*	0.154^*^	0.148^***^	0.103^*^	0.136^***^	0.093^*^
	(0.083)	(0.063)	(0.072)	(0.068)	(0.052)
Constant	1.803^**^	1.294^***^	1.538^**^	1.685^***^	1.416^***^
	(0.207)	(0.162)	(0.127)	(0.286)	(0.156)
District effects	Control	Control	Control	Control	Control
Time effect	Control	Control	Control	Control	Control
Sample size	217	217	217	217	217
*R* ^*2*^	0.482	0.466	0.571	0.493	0.539

***, ** and * represent significant levels of 1%, 5% and 10%, respectively.

Data source: calculated.

Column (1) of Tables 4–6 shows the baseline regression, in which all ITCs pass the significance test and there is a positive relationship with GTFP. According to the regression results, before the inflection point of the “U” shape, for every standard deviation change in technical efficiency, the level of GTFP of smart cities will change by 1.123 times; after the inflection point of the “U” shape, for every standard deviation change in technical efficiency, the level of GTFP of smart cities will change by 0.473 times. The *R*^*2*^ value is 0.604, which indicates that the study sample can explain the real change in GTFP at the level of 60.4%. This indicates that the increase in ITC input enhances GTFP. Moreover, ITC2 also passes the significance test, i.e., there is a “U”-shaped relationship between information technology and communication productive capital investment and GTFP in smart cities. This means that the information technology and communication productive capital input in the early stage of the establishment of smart city leads to the decline of GTFP, which is consistent with the conclusion of theoretical hypothesis 1. With the continuous increase of information technology and communication productive capital investment in the construction of smart cities, the GTFP turns from a decline to an increase. This result verifies the theoretical hypotheses 2 and 3. Therefore, Tables 4–6 again verify the scientific validity of the three theoretical hypotheses proposed in this paper. The above results are consistent with the study by Wang et al. [1] who believe that technological efficiency is the key reason why smart cities affect the level of GTFP. The above results are inconsistent with the studies of Jiang et al. [85] and Wu and Wang [86]. The former takes China’s first batch of smart cities as an example and finds that smart cities have mainly improved their GTFP level through the advancement of green technology. The latter study found that smart cities have significantly improved the low-carbon technology efficiency level of pilot cities, thereby increasing their carbon total factor productivity. The main difference mentioned above is since most existing studies have focused on the long-term effects of smart cities, only focusing on the linear impact of smart cities on technological efficiency and progress, while ignoring the potential nonlinear laws inherent in them. And the above research mainly starts from the first batch of smart cities, ignoring the differential characteristics of the second and third batches of smart cities. In addition, the results of columns (2) - (5) of Tables 4–6 verify the robustness of the model.

#### Comparison of GTFP between cities in the experimental group and the control group.

Although the use of both Malmquist index and panel data model obtained that the GTFP of smart cities experienced a brief decrease in the early stage of construction and then gradually increased, these two methods reflect the average trend of GTFP change in smart cities, which hides the relevant characteristics of individual cities. In view of this, this paper applies the common frontier function to analyze the changing law of GTFP in each pilot city during the construction of smart cities.

**Fig 7:**
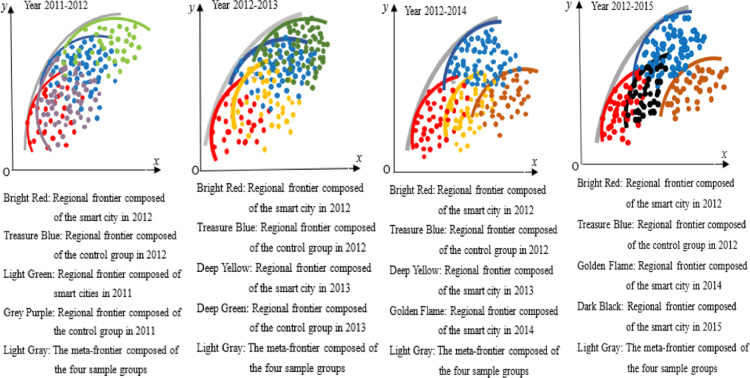
Comparison of GTFP between the first batch of smart city pilots and the control group.

Fig 7 shows the GTFP of the experimental and control groups before and after the implementation of the policies of the first batch of smart city pilots in 2011–2015. The first batch of smart city pilots was established in 2013, and took the year before its establishment, i.e., 2012, as the reference, the four subfigures from left to right are: 2011 and 2012, 2012 and 2013, 2012–2014, 2012 and 2014–2015 for the comparison of GTFP of the experimental group and the control group, respectively. The purpose of choosing 2011 and 2012 as the survey time is to analyze whether there is a difference in GTFP between the experimental group and the control group before the establishment of the smart city; the purpose of choosing 2013–2015 as the survey time is to analyze the change in GTFP of each city in the experimental group and the control group after the establishment of the smart city.

The results show that before the establishment of the smart city, the four regional production frontiers of the experimental and control groups in 2011 and 2012 are on the meta-frontier, i.e., they have the same level of GTFP, as shown in the left 1 subfigure of Fig 7. The experimental and control groups in 2013 and 2012 constructed four regional production frontiers, as shown in the left 2 subfigure of Fig 7. Of these four production frontiers, the regional production frontiers of the 2012 experimental group, the control group, and the 2013 control group form a meta-frontier and are ahead of the regional production front formed by the 2013 smart city. This indicates that one year after the establishment of the smart city, its GTFP declined, not only below the level of the previous year, but also below the level of the control group in the same period. Moreover, 2 years after the establishment of the smart cities, this downward trend is even more obvious, as shown in the left 3 subplot of Fig 7. The regional production frontier formed by the first batch of smart cities in 2014 lags not only the meta-frontier consisting of the experimental and control groups in 2012, but also the regional production frontier formed by the smart cities in 2013. This proves once again that the GTFP in the early stage of smart city construction has a declining trend. That is, hypothesis 1 of this paper is verified. It is worth noting that this downward trend began to reverse in 2015, as shown in the left 4 subplot of Fig 7. Compared with 2014, the regional production frontier formed by the first batch of smart cities in 2015 is closer to the meta-frontier, which indicates that with the gradual improvement of the construction of smart cities, the GTFP begins to improve, i.e., theoretical hypothesis 3 is valid.

**Fig 8:**
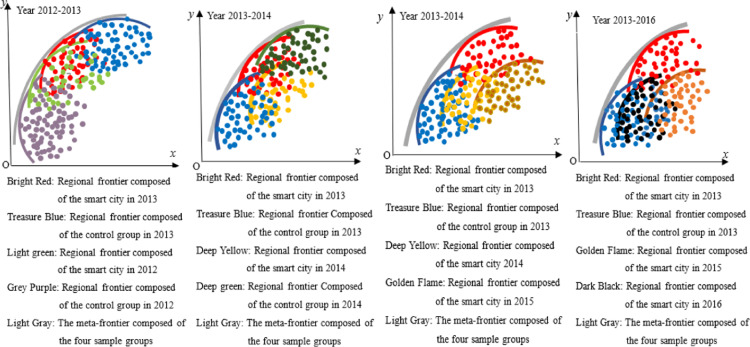
Comparison of GTFP between the second batch of smart city pilots and the control group.

**Fig 9:**
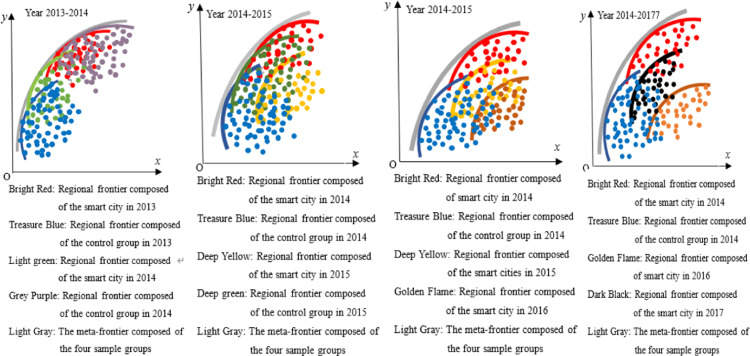
Comparison of GTFP between the third batch of smart city pilots and the control group.

**Fig 10:**
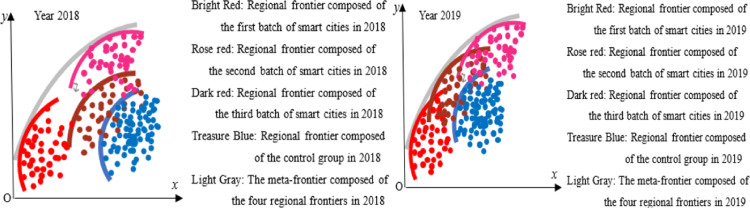
Comparison of GTFP between three batches of smart cities and the control group in 2018 and 2019.

The patterns of change in GTFP for the second and third batches of smart city pilots are similar, as shown in Figs 8 and 9, respectively. Because the second and third batches of smart city pilots were established in 2014 and 2015, respectively, 2013 and 2014 were used as the reference for the two, respectively. The changes in GTFP of the experimental group and the control group between the two years before and after the establishment of these two batches of smart cities and the reference year are compared. They both show that before the establishment of the smart city, the experimental group and the control group have the same level of GTFP, forming a meta-frontier. However, in the 1st and 2nd year after the establishment of the smart city, the GTFP of the pilot city declined consecutively, and this downward trend began to reverse in the 3rd year. This reversal trend is further identified by Fig 10. In the left 1 subplot of Fig 10, all three batches of smart cities in 2018 have higher GTFP compared to the control group. Moreover, all three form a meta-frontier in 2019 and remain ahead of the regional production frontier of the control group, i.e., the left 2 subplot of Fig 10. This result further validates the theoretical hypothesis 3 of this paper.

While the common frontier function reaffirms theoretical assumptions 1 and 3 from a relatively static perspective, note that not all pilot cities in Figs 7–10 are located on the regional or common frontier surface. More eastern smart cities are at or near the common frontier surface, and only a few of the western pilot cities are near or on the frontier surface, compared to all central smart cities which are not on the common frontier surface. This suggests that while the changes in GTFP of smart cities are synchronized to increase, there are large differences in green production capacity. In 2019, for example, most of the smart city pilots in the eastern region are located on or close to the common frontier, with higher productivity and more advanced green technologies; in comparison, only a few smart cities in the western region are located on or close to the common frontier; and the central region’s smart cities have the lowest GTFP among the three regions. This suggests that although smart city construction helps to innovate green technologies, optimize environmentally friendly production processes, and pay more attention to ecological protection, the differences between cities due to their resource endowments, starting points for policy implementation, and management capabilities have led to differences in the level of their green all-important productivity during the construction process. This may be since during the construction of smart cities in the eastern region, each local government played a coordinating role, not only guiding the construction of local digital facilities, but also establishing cooperative relationships with other smart cities to share and build relevant facilities, which not only maximize productivity, but also minimize CO_2_ emissions. The Eastern Digital and Western Computing Project in the western region has helped the western smart cities to improve the utilization efficiency of various resources, relying on their own power resources and natural climate advantages to provide stable data services for the east, and forming a pattern of synergistic economic development with the eastern region. The lack of integrated planning for the construction of smart cities in the central region, and even the excessive construction of computing centers, not only leads to a waste of resource inputs, but also fails to give full play to the role of information infrastructure.

### Research limitations and future research directions

This paper uses the Malmquist index to measure the GTFP of smart cities and further explores its dynamic changes in the short term as well as in the medium and long term, but the study still has the following shortcomings: firstly, the selection of indicators in the Malmquist index model needs to be further improved. This paper draws on the existing indicators used in the evaluation of GTFP and considers the scientific nature and accessibility of the indicators and selects some representative indicators to evaluate the GTFP of the smart city, which can reflect the level of its GTFP to a certain extent. However, due to the availability of data, this paper mainly selects carbon dioxide emissions as the non-desired output and does not consider the emissions of other pollutants. In the future, relevant indicators can be further supplemented and improved to enhance the accuracy of the analysis results. Second, this paper mainly focuses on the impact of China’s smart cities on GTFP and does not analyze the impact of China’s smart cities on GTFP in comparison with smart cities in other regions. This paper mainly takes Chinese smart cities as an example to explore the changes in GTFP of smart cities in the short-term and medium- to long-term. However, the level of smart city construction and economic and social conditions vary greatly in different regions, and their impact on GTFP may have different characteristics and patterns. In the future, further comparisons can be made between the impact of smart city construction on GTFP levels in China and other regions. Finally, this paper mainly analyses the impact of smart cities on GTFP from a macro-regional perspective. However, there may be differences in the GTFP of smart cities for different industries as well as different enterprises, e.g., the trend of change and the mechanism of action may be different in manufacturing and service industries. In the future, the effect of smart cities on GTFP and its mechanism can be further explored from the industry or micro enterprise level.

## Conclusions and suggestion

### Conclusions

Under the trend of global digital transformation and green development, it is of great theoretical and practical significance to explore the impact of smart city construction on the level of GTFP. However, most of the existing studies focus on the long-term effects of smart cities on GTFP, while ignoring the environmental pollution problem caused by large-scale digital construction in the early stages of smart cities. This leads to insufficient understanding of the effect of the implementation of this policy and the inability to develop targeted solutions to this problem. This paper makes up for the above research deficiencies by taking the three batches of smart city pilots in China from 2013–2019 as an example, and using the Malmquist index model, the common frontier function and the panel data method to analyze the pattern of change in the GTFP at the early stage of the construction of China’s smart cities as well as in the mid- to long-term, specifically obtaining the following research conclusions: (1) At the early stage of smart cities GTFP is less than 1, and the overall trend is declining. (2) The GTFP of smart cities shows the law of change of decreasing in the early stage and increasing in the middle and late stage, and the GTFP level in the middle and late stage is greater than 1. (3) Technological efficiency is the key factor for the GTFP of smart cities to show the above changes. Specifically, technical efficiency and GTFP of smart cities show a “U” type relationship. When the smart city is in the middle or late stage of development, for every unit of technical efficiency, the level of GTFP increases by 0.463 units. (4) From the perspective of individual smart cities, the GTFP level of smart cities in the east is the highest, followed by the west and the lowest in the center.

### Suggestion

Based on the above research conclusions, the following suggestions can be obtained:

Firstly, local governments should focus on the long-term benefits of smart city construction and avoid short-sighted behavior. The construction of smart cities is a fundamental and comprehensive system engineering that requires long-term investment, continuous updates, and continuous innovation. Smart cities have certain characteristics of public goods, and their construction should not only enable the market to play a decisive role in resource allocation, but also better leverage the active role of the government in infrastructure construction, innovation platform building, policy environment optimization, and promote the better integration of effective markets and proactive governments. The construction of smart cities should not only rely on buildings to use, but also on using them. It is necessary to encourage the participation of enterprises and all sectors of society, form a situation of multi-party cooperation such as government, enterprises, and scientific research institutions, and attach importance to the cultivation of market entities. Only by truly stimulating market forces can dynamic cumulative effects and benign long-term mechanisms be formed, thus better promoting the green transformation and development of the economy.

Secondly, local governments should pay attention to environmental protection in the early stages of smart city construction. In the early stages of smart city construction, low resource utilization efficiency led to a decrease in GTFP. Therefore, on the one hand, we need to strengthen the construction of new urban infrastructure, deeply promote the cross integration of new generation information technology and urban development, focus on promoting smart applications and green transformation in energy intensive utilization, transportation, waste management, agriculture, forestry, and public management, and improve urban green production capacity and innovation level. For example, establishing green corridors; Building a smart grid and utilizing renewable energy to reduce carbon emissions; Transforming the urban intelligent transportation system, implementing intelligent traffic control systems and intelligent vehicle tracking systems to reduce vehicle congestion; Designing green buildings, with low-carbon emissions and low energy consumption as the main goals in the design and construction of buildings. On the other hand, building a modern government governance system, innovating environmental governance methods, strengthening regional pollution control supervision, guiding enterprises (especially “three high” enterprises) to innovate green technologies, and assisting enterprises in improving resource utilization efficiency. For example, helping enterprises establish waste management systems, introducing waste disposal and reuse technologies. The government plans and designs green cities to reduce energy consumption.

Once again, it will accelerate the transformation speed of information technology and digital technology. Governments at all levels should formulate comprehensive digital and information technology transformation strategies, seize the opportunities brought by the new round of technological revolution and industrial transformation, vigorously promote the construction of smart cities, comprehensively promote the deep integration and development of digital technology with social governance, industrial economy, and green innovation in various fields, enhance the comprehensive application of science and technology, accelerate the circulation and integration of innovative elements, promote industrial transformation and upgrading, better play the technological, configuration, and structural effects of smart cities, stimulate urban green innovation vitality, and promote urban green and sustainable development. Proactively incorporating digital transformation goals into the development strategy formulated by enterprises to accelerate their digital transformation. Strengthen digital governance, establish corresponding systems and standards to ensure the effectiveness, directionality, security, and reliability of digital technology transformation. Intensify policy incentives, attract relevant talents and investments through tax reductions and transfer payments. Encourage information sharing among various industries, reduce information silos, achieve data interoperability and sharing, and reduce obstacles to digital transformation.

Finally, develop a smart city development plan tailored to local conditions and carry out policy pilot implementation work. For the eastern region of China, where the economy is relatively developed, the development of the digital economy is relatively mature. Therefore, it is possible to strengthen the construction of infrastructure such as intelligent transportation and intelligent water supply to improve urban operational efficiency and quality of life. For the relatively underdeveloped western regions of China’s economy, the focus should be on green growth, such as strengthening the development and utilization of renewable energy, increasing investment in ecological protection and restoration, and establishing a smart city ecological management system. At the same time, attention should be paid to the technological progress of non-frontier cities. Build a green technology exchange platform for cities with low GTFP and cutting-edge cities. Cities with poor levels of green technology actively collaborate with cutting-edge cities to develop green technology and strengthen talent exchange between them. Promote the green transformation and low carbon upgrading of non-frontier urban industries and encourage enterprises in these cities to adopt clean production technologies and processes. Establish a technology transfer management organization to build a cooperation platform for both technology supply and demand, accelerate the speed and transfer of technology innovation, and drive more cities to the forefront. Especially optimizing the construction of information infrastructure for smart cities in central China, building cooperation platforms between cities or regions, and reducing redundant construction of facilities.

By exploring the dynamic changes of GTFP in smart cities at different stages and its key factors, this study points out a new direction for subsequent related research. Future research can be carried out from various aspects, on the one hand, to explore the trend and mechanism of GTFP of smart city construction in different regions of the world, and to find out more targeted development strategies to help underdeveloped regions and small and medium-sized cities make better use of smart city construction to improve GTFP; on the other hand, to further study the synergistic effect of smart city construction with other related factors, such as policies, market mechanisms, social culture, etc., and to comprehensively analyze how to enhance GTFP through the synergy of multiple factors. On the other hand, we will further study the synergistic effect of smart city construction with other related factors, such as policy, market mechanism, social culture, etc., and comprehensively analyses how to maximize the promotion of GTFP by smart city through multi-factor synergy, to provide more comprehensive and in-depth theoretical support and practical guidance for the sustainable development of cities.

## Supporting information

S1 Data Dataset. Raw data used in this paper.
(XLSX)
